# Molecular Mechanisms of Transdifferentiation of Adipose-Derived Stem Cells into Neural Cells: Current Status and Perspectives

**DOI:** 10.1155/2018/5630802

**Published:** 2018-09-13

**Authors:** Liang Luo, Da-Hai Hu, James Q. Yin, Ru-Xiang Xu

**Affiliations:** ^1^Department of Burns and Cutaneous Surgery, Xijing Hospital, Fourth Military Medical University, Xi'an, Shanxi 710032, China; ^2^Stem Cell Research Center, Neurosurgery Institute of PLA Army, Beijing 100700, China; ^3^Bayi Brain Hospital, General Hospital of PLA Army, Beijing 100700, China

## Abstract

Neurological diseases can severely compromise both physical and psychological health. Recently, adult mesenchymal stem cell- (MSC-) based cell transplantation has become a potential therapeutic strategy. However, most studies related to the transdifferentiation of MSCs into neural cells have had disappointing outcomes. Better understanding of the mechanisms underlying MSC transdifferentiation is necessary to make adult stem cells more applicable to treating neurological diseases. Several studies have focused on adipose-derived stromal/stem cell (ADSC) transdifferentiation. The purpose of this review is to outline the molecular characterization of ADSCs, to describe the methods for inducing ADSC transdifferentiation, and to examine factors influencing transdifferentiation, including transcription factors, epigenetics, and signaling pathways. Exploring and understanding the mechanisms are a precondition for developing and applying novel cell therapies.

## 1. Introduction

After the groundbreaking studies that succeeded in reprogramming mouse and human somatic cells into induced pluripotent stem cells (iPSCs) [1], researchers have made a great progress in refining reprogramming methods and applying this technology in the clinic to treat human diseases. However, for successful clinical applications, iPSCs must be more efficiently transdifferentiated into different cell types. Furthermore, both embryonic stem cells (ESCs) and iPSCs have potential tumorigenic risks *in vivo* [2, 3], which significantly limits their utility. Lineage-restricted stem cells, such as neural stem cells (NSCs) and adipose-derived mesenchymal stromal/stem cells (ADSCs), do not have this limitation [4, 5]. Recently, a direct reprogramming of one of the cell types into another (transdifferentiation) has become another area of intense study [6]. Transdifferentiation may supplement iPSC technology and avoid the problems of differentiating iPSCs and ESCs into mature cell types. More importantly, this approach would reduce the risk of teratogenesis after incomplete reprogramming and the likelihood of immune rejection and other complications associated with allogeneic transplantations.

Traditionally, nervous system tissue has been considered difficult to regenerate because mature neural cells do not proliferate or differentiate. Consequently, identification of a specific cell capable of neuronal differentiation has generated immense interest. Zuk et al. [7] first found that ADSCs isolated from the adipose stromo-vascular fraction have the capacity for multilineage differentiation. Safford et al. reported that mouse and human ADSCs (hADSCs) could be made to transdifferentiate into neural-like cells [8]. During the past decade, human adipose tissue has been identified as a source of adult multipotent ADSCs, which can transdifferentiate into a range of mesodermal, endodermal, and ectodermal cells [7, 9] in the presence of specific induction factors. These ADSCs have been shown to transdifferentiate into neurons [10, 11], oligodendrocytes [12], and Schwann cells [13]. Therefore, adipose tissue is a likely candidate source of stem cells capable of neural cell transdifferentiation in a short period of time and may potentially strengthen their clinical application. No other tissues appear more practical than adipose tissue, and adequate numbers of ADSCs can easily be isolated and expanded for clinical therapies [14].

Although ADSCs are ideal donor cells for treating neuronal diseases, the outcomes of most *in vivo* ADSC studies have been relatively disappointing. Better understanding of the molecular mechanisms of ADSC transdifferentiation is a key step in optimizing ADSC-neural system therapy. The aim of this review is to discuss the recent literature regarding the molecular mechanisms of ADSC transdifferentiation. We review the epigenetic factors, transcription factors (TFs), and signaling pathways that modulate ADSC transdifferentiation, as well as the development and transdifferentiation of ADSC-derived neural cells.

## 2. Characteristics of ADSCs and NSCs and Methods for Inducing Transdifferentiation

**Figure 1:**
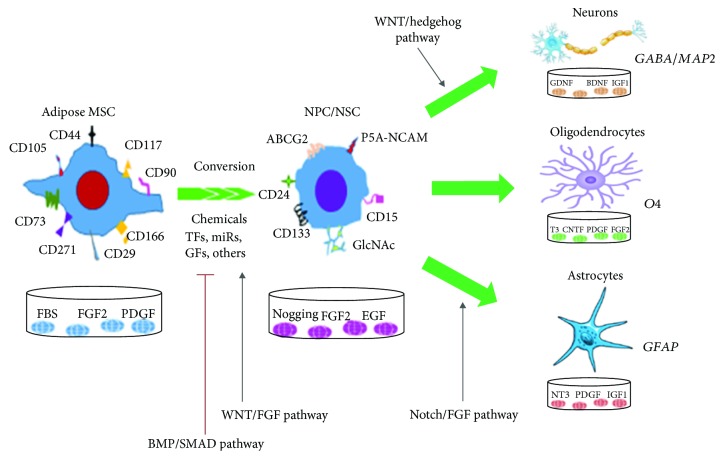
A schematic for the transdifferentiation of ADSCs into NSCs and neural cells, indicating relevant influences such as cell surface markers, transcriptional factors, culture media, and signaling pathways. The details can be seen in the text. TFs: transcription factors; miRs: microRNAs; GFs: growth factors; MSCs: mesenchymal stem cells; PSA-NCAM: polysialic acid neural cell adhesion molecule; GlcNAc: N-acetylglucosamine; PDGF: platelet-derived growth factor; IGF: insulin-like growth factor; CNTF: ciliary neurotrophic factor; GABA: *γ*-aminobutyric acid; GDNF: glial-derived neurotrophic factor; BDNF: brain-derived neurotrophic factor; T3: 3,5,3′-triiodothyronine; NT3: neurotrophin-3.

In 2006, the committee of the International Society for Cellular Therapy established the following minimum criteria for characterizing human mesenchymal stem cells (MSCs), and ADSCs comply with these criteria [15]: (1) the cells should adhere to plastic in culture; (2) more than 95% of them must express CD105, CD73, and CD90 but not express (<2%) CD34, CD45, CD14 or CD11b, CD79*α* or CD19, or HLA-DR molecules; and (3) they should be able to differentiate into osteoblasts, adipocytes, and chondrocytes [16]. Recently, several new markers, such as CD146, CD271, SSEA1/4, and CD44, have been identified, and CD271 has been proposed as one of the most specific MSC markers (Figure 1) [17, 18].

Traditionally, MSCs can be obtained from bone marrow stem cells (BMSCs), but their expansion is limited and the population is small, comprising only 0.01~0.0001% of bone marrow cells in adult individuals [19]. However, ADSCs represent more than 1% of the adipose cell population, producing at least 100 times more MSCs than those from bone marrow [20]. Unlike BMSCs, which are difficult to obtain, adipose tissue biopsies can be obtained by relatively safe, popular liposuction procedures, one of the usual plastic surgeries performed in the United States (http://www.surgery.org) [21]. ADSCs are therefore an attractive source of cells for genetic, cellular, and molecular analyses and for clinical applications. Most neurological diseases, such as nerve injury and neurodegenerative disorders, are due to the loss or dysfunction of neural cells [22]. However, if we can obtain a sufficient supply of NSC/NPCs (neural progenitor cells) from transdifferentiated ADSCs, the problem can be solved to a great extent.

To achieve this purpose, one should first identify NSC/NPCs with relatively definitive markers. Recently, many cell surface and intracellular molecules have been identified: the stage-specific embryonic antigen-1 (SSEA-1/Lewis X/CD15) [23], CD24 [24], p75 receptor [25], ABCG2 [26], brain-specific chondroitin sulfate proteoglycans [27], O-glycans, and PSA-NCAM [28] have been utilized to purify a population of cells from neural tissues (Figure 1). On the other hand, several markers, such as CD133, NESTIN, SOX1/2, PAX6, MUSASHI-1, and VIMENTIN [29], have been often taken as markers to identify *in vitro* NSC-like cells derived from other types of cells.

The evaluation methods for transdifferentiation of ADSCs into NSCs measure the colony formation efficiency (CFE), induced conversion efficiency, and total conversion time. The estimates of neural stem cell derivation efficiencies obtained by different induction methods are summarized in Tables 1 and 2. One may conclude that most studies claim that the conversion efficiency of ADSC transdifferentiation into NSCs is very high (>10%) and that the conversion time is short (<14 d). However, these so-called high-efficiency methods have not been rigorously scrutinized, and most of these methods have not provided the colony formation efficiencies. Therefore, we think that the majority of “NSCs” reported in these articles were probably not NSCs or NPCs but rather were mostly NSC-like cells, which are like an intermediate-state cell that is a type cell of the intermediate process of transdifferentiating from ADSCs into NSCs. In contrast, some inefficient methods, such as those reported by Cairns and his colleague, may represent the true efficiency achieved so far [30] (Table 1); they reported that the CFE was 0.01% during the 30-day induced conversion from ADSCs to NSCs, for which they used a classic induction method using OSKM transcription factors.

Some reports have shown that somatic cells, such as mouse or human fibroblasts, can directly transdifferentiate into functional neurons [31, 32]. However, in the studies of ADSC transdifferentiation into neural cells, the data provided only weak evidence and indirect observations, such as cell polarity and relevant protein marker expression at appropriate locations. Few studies have strictly demonstrated that ADSCs can generate functional neurons; in most cases, the reported results rely too much on the morphological changes and/or neuronal marker expression as part of the cell identification criteria. Overall, researchers must provide more convincing proof of neuronal transdifferentiation, including depolarization, synapse formation and function, and a delayed-rectifier type of K^+^ and Na^+^ current. If transplanted, the transdifferentiated neurons must also contact and communicate with other neural cells. Furthermore, behavioral experiments should be conducted after transplantation.

The ultimate goal of ADSC use is to generate the cell population of interest for clinical transplantation. For ADSCs to become ideal for neurological disease therapy, they must generate a sufficient number of functional and high-quality neural cells. To this end, there are three approaches: (1) directed induction of ADSCs to neural cells; (2) first, induction of ADSCs to NSCs and then induction of those into other neural cells; and (3) conversion of ADSCs to iPS cells and induction of those into neural cells. At a first glance, method (1) appears to be the best, but it has not yet produced fully functional neural cells. Another drawback of method (1) is that the induced nerve cells do not proliferate. Method (3) has been developed with forced expression of defined factors using multiple viral vectors. However, such iPS cells contain a large number of viral vector integrations, which may cause unpredictable genetic dysfunction. Thus, a comprehensive consideration of these factors suggests that method (2) may be the best of the three.

In summary, combinations of TFs, small molecules, nutrients, and cytokines can induce ADSCs to transdifferentiate into neural-like cells (Tables 1 and 2). Furthermore, there are still some problems in validating the method for inducing transdifferentiation of ADSCs: few related studies of ADSC transdifferentiation to neural cells were conducted *in vivo*, and most of these studies have not included functional assessments, such as electrophysiology; therefore, the optimal combination of factors remains to be established.

## 3. Epigenetic Regulation of Transdifferentiation of ADSCs into Neural Cells

Epigenetic factors are known to play a pivotal role in determining stem cell fate and differentiation. These factors include chromatin remodeling, histone modification, DNA methylation, and noncoding RNA regulation. At present, there are challenging problems to solve in transdifferentiation of ADSCs, and the key to solving these problems is to achieve an in-depth understanding of epigenetic mechanisms of transdifferentiation.

Transdifferentiation of cells is accompanied by drastic changes in gene expression and epigenetic profiles. MSC transdifferentiation into neural cells should include 2 major events: (1) the disruption of the apparent steady state of the original cell's epigenetic modification and (2) the establishment of homeostasis of NSCs or neural cell-specific modifications. ADSCs are also strictly guarded by an epigenetic barrier, and they acquire more pluripotency by crossing that barrier with the help of relevant reprogramming factors of neural cells, which include several key transcription factors (TFs) [65]. Epigenetic researchers focus on covalent and noncovalent modifications of DNA and histones and the mechanisms by which such modifications affect chromatin structure and gene expression. Currently, a limited number of published studies of ADSC transdifferentiation mainly focus on histone modification, DNA methylation, and noncoding RNA regulation.

### 3.1. Histone Modification

Histone posttranslational modifications include methylation, acetylation, phosphorylation, ubiquitylation, and other translational modifications of the tail end sites of the core histones [66]. The histone modification mechanisms underlying the transdifferentiation of ADSCs into neural cells are largely unknown. So far, a few papers have only focused on histone acetylation and methylation research.

Histone acetylation is one of the most abundant and dynamic histone modifications [67]. Generally, acetylation of histone tails represents a major regulatory mechanism during gene activation and repression. Actively transcribed regions of the genome tend to be hyperacetylated, whereas inactive regions are hypoacetylated.

Histone acetylation weakens the interaction between histone tails and DNA, which creates a space for factors that bind to the promoter regions and initiate gene transcription, and p300/CBP is also believed to be involved in the processes of MSC transdifferentiation [68, 69]. For example, during neurogenesis, Ngn1 binds to P300/CBP, which prevents differentiation into glial cells [70]. In contrast, the histone deacetylase (HADC) inhibitors TSA, VPA, MS-275, and NaB could induce neurogenic differentiation of hADSCs, as shown by RT-PCR and Western blot analysis, and most neuronal marker genes were expressed when neural-induced hADSCs were treated with the HDAC inhibitors individually. Furthermore, studies also discovered that expression of most Wnt-related genes was highly increased following treatment with the HDAC inhibitors. In short, the HDAC inhibitors could induce neurogenic differentiation of hADSCs by activating the canonical Wnt or noncanonical Wnt signaling pathways [71]. Another study also reports that histone deacetylase inhibitor valproic acid (VPA) enhances the neural differentiation of mesenchymal stem cells into neural cells. During MSC differentiation, histone deacetylase, HDAC2, is reduced in the VPA set, whereas HDAC1 remains unchanged [72]. Moreover, during human MSC differentiation, the Sox9 transcriptional apparatus activates its target gene expression through p300-mediated histone acetylation of chromatin. These findings suggest that lineage-specific transcription factors can interact with chromatin and activate associated transcription via regulation of chromatin modification [73]. Based on the above and previously published epigenetics studies, in general, a more global level of histone acetylation rather than any specific residue is critical [74].

In contrast to acetylation, there is a clear functional distinction between histone methylation marks, concerning both the exact histone residues and their degree of modification [75]. Thus, H3K9me3 and H4K20me3 are enriched near the boundaries of large heterochromatic domains, and H3K9me1 and H4K20me1 are found primarily in active genes [76]. It has been reported that lysine methylation is responsible for the transcriptionally silenced or active chromatin status, whether it occurs at H3K4, H3K9, H3K27, H3K20, H3K36, or H3K79 residues [66]. During neurogenic transdifferentiation of ADSCs, dynamic changes are observed in methylation of histones H3K4, H3K9, and H3K27 in the NES locus [49].

Taken together, these studies provide an insight into the epigenetic mechanisms of ADSC transdifferentiation into neural cells and suggest molecular models of how the key factors are linked to histone modifications in ADSCs. Histone acetylation/deacetylation and methylation/demethylation exist simultaneously in the process of transdifferentiation, and they closely link and regulate the entire transdifferentiation process, but most of the specific mechanisms of histone modification remain to be elucidated in ADSC transdifferentiation.

### 3.2. DNA Methylation

DNA methylation is a crucial epigenetic mechanism and is essential for normal cellular functions and development, especially for the imprinting of specific genes, X chromosome inactivation, and cell type-specific gene expression [77]. DNA methylation typically occurs in a CpG dinucleotide context. A methyl group is added to cytosine within a CpG dinucleotide by DNA methyltransferases (DNMT) DNMT1, DNMT3a, and DNMT3b [78], and the status of CpG methylation in the genomes of ADSCs reflects their transdifferentiation potential [79].

Mesenchymal stem cells have the potential to transdifferentiate into NSCs or other neural cells. Changing the methylation status of lineage-specific genes may be a key step in the processes of neural cell generation. Using inhibitor and activator agents of DNA methylation and acetylation, scientists found that MSCs can be induced to express high levels of neural stem cell marker SOX2. Exposing these modified cells to a neural environment promoted efficient generation of neural stem-like cells as well as cells with neuronal and glial characteristics [80]. Studies found that the neural-specific enhancer regions of Nestin are demethylated during reprogramming and remethylated upon neurogenic differentiation [49].

On the other hand, attenuation of adipogenesis may be a key process during the transdifferentiation of ADSCs into neural cells. A nuclear hormone receptor, peroxisome proliferator-activated receptor-gamma (PPAR-*γ*), plays a crucial role in adipogenesis, in which TFs with chromatin remodeling activities sustain the role of epigenetic regulation [81]. Noer et al. analyzed the DNA methylation profiles of both adipogenic and nonadipogenic gene promoters in ADSCs. Studies in freshly isolated ADSCs found that adipogenic gene (PPAR-*γ*2, leptin, FABP4, and LPL) promoters appear to be globally hypomethylated, whereas myogenic and endothelial cell regulatory regions tend to be more methylated [82]. However, in general, due to very few ADSC epigenetic studies, key methylation mechanisms in transdifferentiation of ADSCs into NSCs are still largely unknown.

### 3.3. Noncoding RNA Regulation

During cell differentiation, multiple genes must be expressed coordinately at precise levels, both spatially and temporally. Feedback and feedforward pathways are key regulatory strategies for maintaining this coordination. MicroRNAs are essential mediators in feedback and feedforward regulation.

Recently, miR-124 was found to be significantly upregulated during neurogenic transdifferentiation of ADSCs, and knockdown of miR-124 blocked ADSC neurogenic transdifferentiation. miR-124 modulates neurogenic transdifferentiation, in part, via the RhoA/ROCK1 signaling pathway [83]. Furthermore, ADSCs were transduced by lentiviral vectors containing miRNA-34a as the way to regenerate the sciatic nerve in a surgically induced sciatic nerve injury rat model. The results showed that transplantation of miRNA-34a-overexpressing adipose-derived stem cells significantly enhanced the restoration of nerve continuity and functional recovery [84].

Relatively few miRNAs were reported to be involved in ASDC transdifferentiation compared with those in studies of NSCs, so we summarized miRNAs associated with ASDC differentiation and antiadipogenic genes (Table 3); additionally, we list NSC-specific miRNAs in Table 4 for reference.

## 4. Key Transcription Factors Involved in ADSC Transdifferentiation

In 2006, the Yamanaka group showed that mouse fibroblast cells can be reprogrammed into iPSCs by overexpression of OCT4, SOX2, KLF4, and cMyc (OSKM) TFs [1]. Since then, many groups have studied the methods and mechanisms of the somatic cell reprogramming process by analyzing epigenetic and transcriptional changes at different time points after factor induction in different somatic cells. It has been reported that OSKM can reprogram ADSCs to iPSCs [104, 105].

To date, there have only been a few reports on ADSC transdifferentiation by TFs. After being transfected with TFs OCT3/4, SOX2, KLF4, and c-MYC and then further treated with neural-inducing medium, hADSCs switched to transdifferentiation toward neural cell lineages [40]. ADSCs can be converted into induced NSC-like cells with a single transcription factor, SOX2 [38]. Using a 3-step NSC-inducing protocol, highly purified NSCs can be derived from hADSCs by SOX1 activation [35]. Expression patterns of key transcription factors, such as PAX6, MASH1, NGN2, NeuroD1, TBR2, and TBR1, were changed during neurogenic transdifferentiation of hADSCs [60]. In general, relevant ADSC transdifferentiation research has been infrequently reported.

Although few transdifferentiation studies use ADSCs as a cell model, some elegant studies have detailed TF transfections and reprogramming methods, in which fibroblasts, which originate from the mesoderm, differentiate into neural cells or NSCs. These TFs include (but are not limited to) the following: SOX2, PAX6, BRN2 or BRN4, NG, ASCL1 and MYT1l, Nr2e1 (TLX), BMI1, FOXG1, and E47/TCF3 [106]. It is reasonable to suggest that these TFs may be essential for transforming ADSCs to neural cells by changing relevant epigenetic modifications or initiating specific programs. These findings also hint that overexpression of a few key factors can drive ADSCs to transdifferentiate directly into neural cells.

## 5. Signaling Pathways Implemented in ADSC Transdifferentiation

**Figure 2:**
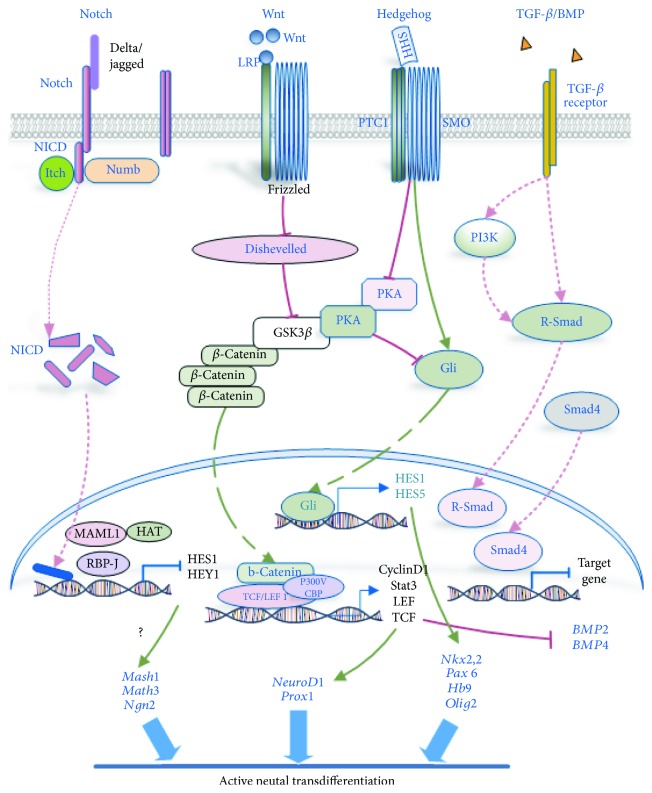
Overview of several important pathways involved in regulating the transdifferentiation of NSCs and neural cells. The Wnt, Notch, hedgehog, and TGF-*β* signaling pathways have been implicated in the transdifferentiation of neural cells. Activation or inhibition of these signaling pathways as well as their cross-talk may initiate cell conversion, maintain the self-renewal of stem cells, and drive their transdifferentiation. Akt: protein kinase B; Dvl: dishevelled; GFs: growth factors; GliR: Gli repressors; GSK3*β*: glycogen synthase 3 beta; LEF1: lymphoid-enhancing factor-1; NICD1: Notch intracellular domain-1; PI3K: phosphatidylinositol-3-kinase; PKA: protein kinase A; Ptch: patched; R-smad: receptor-regulated Smads; Shh: sonic hedgehog protein; SMO: smoothened; TCF: T cell factor transcription factor; Wnt: wingless.

During transdifferentiation into neural cells, ADSCs are stimulated by xenobiotics or specific factors and the corresponding signaling pathways and TFs are activated, resulting in the partial methylation or acetylation of genomic regions and activation of further transdifferentiation processes. Below, we review the crucial signaling pathways in the transdifferentiation of ADSCs to neural cells (Figures 1 and 2).

### 5.1. WNT and *β*-Catenin Pathway

WNT proteins are a class of highly conserved glycoproteins with key roles in cell development and differentiation [107]. Activation of WNT/*β*-catenin signaling accelerates the transdifferentiation of MSCs while depressing commitment to the adipocytic lineage [108]. WNT signaling regulates adipocyte differentiation by repressing the expression of CEBP*α* and PPAR-*γ*, the central regulators of adipocyte differentiation. Recently, it was observed that WNT/*β*-catenin signaling was activated during the transdifferentiation of hADSCs into neural cells [35, 109]. Wnt5a promoted hADSC transdifferentiation into neural cells, binding to the Fz3/Fz5 receptor, and signaling by the Wnt5a-JNK pathway [109]. The expression of genes downstream of the WNT/*β*-catenin pathway, such as cyclin D1 and Stat3, increased [110], while BMP2 and BMP4 expression decreased during early differentiation [111]. Genetic studies have established that activated WNT/*β*-catenin signaling is crucial for neural cell development [112].

Moreover, the WNT/*β*-catenin pathway probably regulates NSC maintenance and differentiation throughout development [113]. In the WNT/*β*-catenin pathway, nonphosphorylated *β*-catenin is expressed in the NSC cytoplasm, then translocates to the nucleus and binds to the LEF/TCF TFs, and then activates the transcription of downstream genes, such as Neurod1 and Prox1, which are TFs specifically involved in neuronal differentiation [114]. Another study indicated that constitutive activation of the Wnt/*β*-catenin pathway in NSCs disrupted the proliferation and migration of neurons within the CNS [115]. Therefore, it is possible that the WNT/*β*-catenin pathway must be tightly controlled in a time- and cell type-specific manner. In short, activation of WNT/*β*-catenin signaling plays a crucial role in promoting the transdifferentiation of ADSCs towards a neural fate.

### 5.2. Notch Pathway

The Notch signaling pathway is highly conserved and exists in all vertebrates [116]. In hADSCs, Notch signaling maintains stem cell self-renewal and inhibits the differentiation into adipocytes [117]. If the Notch pathway is downregulated, hADSCs will transdifferentiate in many directions into cells including neural cells [118, 119], osteocytes [120], and other cell types. The type of transdifferentiated cells will be decided by the inducing environment. Notch is also a key regulator of cell transdifferentiation. Previous reports have indicated that Notch signaling occurs in proliferating hADSCs and is downregulated when cells are transdifferentiated to a neuronal phenotype [119]. On the other hand, Notch was found to be required for the expansion and self-renewal of NSCs in vitro and in vivo [121], and this signaling pathway is also a key regulator of stem cell lineage commitment and differentiation [121]. Notch receptor activation induces expression of the specific target genes hairy and enhancer of split 3 (HES3) and sonic hedgehog (Shh) through rapid activation of cytoplasmic signals, including Akt and STAT3, and promotes NSC survival [122]. These results indicate that Notch signaling affects NSC expansion *in vitro* and *in vivo*. Future studies will provide novel insights into how Notch accurately regulates ADSC transdifferentiation into neural cells and will elucidate common mechanisms of the Notch pathway regulation.

### 5.3. TGF-*β* and BMP Signaling

The transforming growth factor-*β* (TGF-*β*) superfamily comprises the TGF-*β*/activin/nodal and the bone morphogenetic protein (BMP) subfamilies. TGF-*β* family proteins are bifunctional regulators of proliferation or differentiation of stem cells [123]. Signaling gradients, activated by the BMPs, often generate alternative differentiation pathways.

The TGF-*β* family proteins are prototypes of multifunctional growth factors and control switches in regulating key events in hADSC and NSC proliferation, transdifferentiation, migration, and apoptosis [124]. The effects of BMP signaling on NSCs change with developmental stages and are varied. Some studies have identified a BMP signaling inhibitor, Noggin, that can lead to efficient generation of NPCs from human pluripotent cells [125]. Moreover, BMP2 is overexpressed in both type 1 and type 2 astrocytes, but it has no detectable expression in neurons and oligodendrocytes, which indicates that astrocytes may be a source of BMPs during NSC differentiation [126]. BMP5/7 is a regulator of neural stem cell development into mDA neurons in the brain [127] and is involved in neural induction through an interaction with calcineurin-regulated Smad1/5 proteins [128]. These studies indicate that the precise function of the BMP protein subfamily likely depends on the cell context-dependent signaling network.

In brief, BMP and TGF-*β* activate or inhibit cell proliferation, apoptosis, and differentiation. These seemingly contradictory TGF-*β* superfamily functions can be attributed to the level of gene expression, the cross-talk between TGF-*β*/Smad and other signaling pathways (Figure 2), and the stimulation of different TFs that influence the signaling pathways.

### 5.4. Sonic Hedgehog Pathway

Sonic hedgehog receptors consisting of patched (Ptch) and smoothened (SMO) are important in regulating vertebrate organogenesis. The Shh pathway controls cell division and maintains functions of stem cells. In ADSCs, the Shh pathway is involved in the maintenance of stem cell properties and decreases in proliferation during differentiation [51]. Moreover, Shh influences hADSC transdifferentiation during neurogenesis. Previous reports have shown that all hADSCs have the capacity for an active hedgehog pathway through expression of genes that are inhibited after neuronal induction [129]. Shh was often used with RA in induction medium during neural induction from hADSCs. One study showed that neuron-like cells were obtained from hADSCs by activating Shh, RA, and MAPK/ERK signaling and the neuron-like cells expressed the Nkx2.2, Pax6, Hb9, and Olig2 gene [130]. Using *in vivo* genetic fate mapping, both quiescent NSCs and transit-amplifying progenitor cells in the subventricular zone and subgranular zone were shown to respond to Shh signaling and contribute to the ongoing neurogenesis in the adult forebrain [131]. These results suggest that the Shh pathway directs lineage transdifferentiation of ADSCs and is likely involved in neuronal transdifferentiation of ADSCs (Figure 1).

## 6. Challenges and Issues for Transdifferentiation of ADSCs into Neural Cells

Ample evidence suggests that the ADSC is an ideal cell for regenerative medicine and immunosuppressive cellular therapies. However, to date, few groups have provided clear evidence that ADSCs can transdifferentiate into mature or functional neuronal cells *in vivo* or *in vitro*. Expression of a delayed-rectifier type of K^+^ current would indicate a more functional neuronal phenotype. So far, there has been no demonstration of neuronal depolarization or synaptic functioning in transdifferentiated cells cultured *in vitro.* The main reasons for this lack of evidence are the following challenges in ADSC transdifferentiation:

1. ADSCs constitute a heterogeneous population, which itself is a challenge for ADSC transdifferentiation. ADSCs from different donors have different characteristics, including age of the cell donor and use of fat from different parts of the body, which could affect the reproducibility of experiments. Another consequence of ADSC heterogeneity may be the presence of other stem cell types in the isolated adipose tissues. More importantly, there could be some problems with current induction methods, and ADSCs have never been completely converted into true neural cells because one or more programs specific for natural neural cells have not been activated.
2. Until now, there has been no single, universal ADSC marker and no specific neural or NSC marker. The lack of a specific ADSC marker means that there is no way to obtain a highly purified ADSC population. The heterogeneity of ADSC populations combined with different protocols of cell isolation and expansion restricts the ability to precisely analyze and identify specific properties of stem cells. Similarly, because of a lack of specific neural markers, it is difficult to assess the results of ADSC transdifferentiation into NSC, which should be based on 2 or more types of markers, such as a combination of a surface marker and a TF marker (e.g., Nestin, Pax6, and Sox2).
3. Under normal culture conditions, ADSCs can spontaneously express some neural markers [132] or change morphology and related neural marker expression levels [133, 134]. This phenomenon requires further studies to elucidate the relevance of markers or morphology to ADSC transdifferentiation.
4. For the induction of ADSCs to NSCs, some studies only used immunocytochemistry or flow cytometry methods to identify whether ADSCs transdifferentiate into NSCs. We recommend that the assessment of ASC transdifferentiation into NSCs must use colony formation efficiency to avoid false-positive results due to the reasons mentioned above.
5. In most publications, the majority of methods for measuring the induction efficiency use marker expression of NSCs and neural cells. Some studies do not even provide the statistical data of multiple sets of experiments. For the reasons mentioned in 2, we recommend using more than three well-recognized antibodies/markers to verify or assess the differentiation efficiency. In addition, due to the popularity of whole-genome sequencing and cost reduction, we recommend using RNA-seq to assess the quality of differentiation.
6. Up to now, ADSCs have directly been used in many therapeutic studies and clinical trials, and the majority of these studies and trials used nontransdifferentiated cell types. Clearly, cell therapy of ADSCs transdifferentiated to functional neural cells should be more effective for neurological disorders; however, to improve the efficiency of clinical-grade ADSC transdifferentiation and to provide sufficient number of high-quality clinical transdifferentiated cells in a short time, we must face these challenges squarely when the relevant technologies are applied to clinical therapy.

Ultimately, we must do more experiments to establish a strict control of cell differentiation and more rigorous work to verify our hypothesis.

## 7. Conclusion

It would be a mistake to conclude that a functional neuron has been obtained solely based on observing a neural-like morphology or the expression of several neuronal markers during transdifferentiation. Instead, we must do more to validate neural cell function. Genuine neural cell differentiation should yield full cell functionality, which can be demonstrated through the expression of transcriptomes of neuronal genes and electrophysiology.

Neural cells can be generated from MSCs, but current approaches show low efficiency and are complex. No convincing method for the directed transdifferentiation of human ADSCs toward functional neural cells has been reported. The current situation severely limits the usage of these cells as a model for tissue engineering or cell therapy.

In conclusion, several tasks should be addressed in future studies:

- To clarify the molecular mechanisms underlying ADSC transdifferentiation into NSCs
- To verify the function of neurons induced from ADSCs more strictly, using a variety of methods to verify the existence of K^+^ and Na^+^ ion channels and the establishment of synaptic networks after transplantation
- To require better characterization, including a clear definition of a set of markers determining ADSCs and NSCs
- To develop better methods for inducing the transdifferentiation of ADSCs into functional NSCs on a clinical scale
- To investigate the safety of ADSC-derived NSCs and their descendant neural cells in patients

We hope that in the near future, new methods for inducing transdifferentiation will improve the existing ADSC transdifferentiation techniques.

## Figures and Tables

**Table 1:** List of transdifferentiation efficiency of ADSCs into NSCs.

Classification	Induction method	Duration	Efficiencies	CFE	Evaluation methods	Authors (year)
Growth factors and cytokines	B27, EGF, FGF	10–20 days	47.6~71.2%	<54% colony	ICC (Nestin, Fibr), qRT, EPA	Hermann et al. (2004) [33]
	B27, EGF, FGF	8–11 days	0.79%	Not mentioned	ICC/FCM (MAP2ab, GFAP, CD133), RT	Kang et al. (2004) [34]
	N2, B27, BME, NEAA, bFGF, EGF	22 days	>95%	Not mentioned	ICC, qRT, EPA	Feng et al. (2014) [35]
	B27, EGF, FGF	6 days	~15.4%	Not mentioned	ICC (Ki67, Nestin)	Yang et al. (2015) [36]
	B27, EGF, FGF	7 days	>80%	Not mentioned	ICC (Nestin, Sox2, Map2, NF-68)	Darvishi et al. (2017) [11]
	B27, N2, bFGF, EGF	7–10 days	1/1 × 10^–7^	1/1 × 10^–7^	ICC (Sox2, Nestin, Tuj1), qRT, EPA	Petersen et al. (2018) [37]

Small molecular & growth factors	SB431542 (SB), LDN193189 (L), noggin (N)	20 days	>85%	Not mentioned	FCM (NCAM, Nestin, Ki67)	Park et al. (2017) [10]

Transcription factors	OCT4, KLF4, SOX2, c-MYC	30 days	0.01%	0.01%	ICC (Sox1, Sox2, Nestin, Pax6, CD133, Ki67), EPA, TEA	Cairns et al. (2016) [30]
	Sox2	14 days	—	Not mentioned	ICC (Sox2, Pax6, Nestin)	Qin et al. (2015) [38]

Others	Lentivirus-GFP	10 days	—	Not mentioned	ICC (Nestin, NeuN, GFAP)	Zhang et al. (2014) [39]

ICC: immunocytochemistry; qRT: quantitative real-time polymerase chain reaction; EPA: electrophysiology assay; RT: reverse transcription; TEA: tissue engineering assay; CFE: colony formation efficiency.

**Table 2:** List of protocols inducing the transdifferentiation of ADSCs into neural cells.

Class	Factors	Species of ADSCs	Targeted cell type	References
Transcription factors	OSKM	Human	NPCs, NCs	[40]
	Sox2	Mouse	NSC-like cells	[38]
	Nurr-1	Rat	NCs	[41]

Growth factors and cytokines	bFGF and EGF	Human/mouse/rat	NSCs, NCs	Almost all references
	PDGF	Human/mouse/rat	NSCs, NCs	[9, 35, 42]
	BDNF	Human/mouse/rat	NSCs, NCs	[11, 43–48]
	LIF	Human	Schwann-like cells	[46]
	Heregulin-beta	Human	Schwann-like cells	[42]
	GGF-2	Rat	NCs	[9]
	GDNF	Rat	NCs	[11, 45]
	CNTF	Rat	NSCs, neurons	[11]
	NT-3	Rat	NSCs, neurons	[11, 44, 48]

Small molecules (epigenetic)	VPA	Mouse/human	NCs	[8, 49]
	SB431542/dorsomorphin	Human	Neurons	[50]

Signaling factors	Retinoic acid	Human/mouse/rat	NSCs, NCs	[11, 35, 40, 45, 47, 51–53]
	Forskolin	Human/mouse/rat	NSCs, NCs	[8, 9, 45, 46, 54]
	cAMP	Human	NCs	[49]
	IBMX	Human/mouse/rat	NSCs, NCs	[43, 49, 55, 56]

Hormones	Hydrocortisone	Mouse	NCs	[8]
	Dexamethasone	Rat	Schwann-like cells	[55]
	Insulin	Human/mouse/rat	NSCs, NCs	[8, 43, 45, 55, 56]
	Indomethacin	Human/mouse/rat	NSCs, NCs	[43, 55, 56]

Other factors	Conditioned medium	Human	NCs	[57]
	Rat sciatic nerve leachate	Rat	Schwann-like cells	[55]
	Alginate hydrogel	Human	Neurons	[58]
	Electrical stimulation	Rat	NCs	[59]

^∗^Controversial chemical	BHA (butylated hydroxyanisole)	Human/mouse/rat	NSCs, NCs	[8, 45, 51, 60]
	BME (2-mercaptoethanol)	Human	NCs	[51]
	BHA/BME/DMSO/	Human/mouse/rat	NCs	[7, 61–63]

^∗^The protocol to induce neural transdifferentiation of ADSCs using some chemical (such as DMSO, BHA (butylated hydroxyanisole), and BME (2-mercaptoethanol)) has been questioned by many researchers [64], so we list these items separately.

**Table 3:** miRNAs associated with differentiation and antiadipogenic effects.

miRNA	Target	References
miR-22	*HDAC6*	[85]
miR-27a/b, miR-130	PPAR	[86]
miR-138	*EID1*	[87]
miR-145	*KLF4*	[88]
miR-155	*LEBPA* and *CEBPB*	[89]
miR-215	*FNDC3B* and *CTNNBIP1*	[90]
miR-224	*EGR2* and *ACSL4*	[91]
miR-369-5p	*FABP4*	[92]
miR-375	*ADIPOR2*	[93]

**Table 4:** Neural stem cell- or neural cell-specific microRNAs.

miRNA	Effect on NSCs or neural cells	Target(s)	Ref.
miR-9	Neural stem cell self-renewal	TLX (NR2E1), REST, FoxG1, Her5, Her9	[94, 95]
miR-137	Promotion of proliferation and repression of differentiation	Ezh2, PcG, MeCP2	[96, 97]
let-7b	Inhibition of NSC proliferation and accelerated neural differentiation	Hmga2	[98–100]
miR-184	Promotion of neural stem cell proliferation and inhibition of differentiation by targeting Numb-like	MBD1	[101]
miR-124	Neuronal differentiation	REST (NRSF), PTBP	[102]
miR-132	Radial-glial stem cell self-renewal	CREB, Nurr1	[103]
miR-138	Synaptic plasticity	Lypla1	[103]

## References

[1] Takahashi K., Yamanaka S. (2006). Induction of pluripotent stem cells from mouse embryonic and adult fibroblast cultures by defined factors. *Cell*.

[2] Lee A. S., Tang C., Rao M. S., Weissman I. L., Wu J. C. (2013). Tumorigenicity as a clinical hurdle for pluripotent stem cell therapies. *Nature Medicine*.

[3] Tao H., Chen X., Wei A. (2018). Comparison of teratoma formation between embryonic stem cells and parthenogenetic embryonic stem cells by molecular imaging. *Stem Cells International*.

[4] Barkholt L., Flory E., Jekerle V. (2013). Risk of tumorigenicity in mesenchymal stromal cell–based therapies—bridging scientific observations and regulatory viewpoints. *Cytotherapy*.

[5] Mohr A., Zwacka R. (2018). The future of mesenchymal stem cell-based therapeutic approaches for cancer - from cells to ghosts. *Cancer Letters*.

[6] Cieslar-Pobuda A., Knoflach V., Ringh M. V. (2017). Transdifferentiation and reprogramming: overview of the processes, their similarities and differences. * Biochimica et Biophysica Acta (BBA) - Molecular Cell Research*.

[7] Zuk P. A., Zhu M., Ashjian P. (2002). Human adipose tissue is a source of multipotent stem cells. *Molecular Biology of the Cell*.

[8] Safford K. M., Hicok K. C., Safford S. D. (2002). Neurogenic differentiation of murine and human adipose-derived stromal cells. *Biochemical and Biophysical Research Communications*.

[9] Kingham P. J., Kalbermatten D. F., Mahay D., Armstrong S. J., Wiberg M., Terenghi G. (2007). Adipose-derived stem cells differentiate into a Schwann cell phenotype and promote neurite outgrowth in vitro. *Experimental Neurology*.

[10] Park J., Lee N., Lee J. (2017). Small molecule-based lineage switch of human adipose-derived stem cells into neural stem cells and functional GABAergic neurons. *Scientific Reports*.

[11] Darvishi M., Tiraihi T., Mesbah-Namin S. A., Delshad A., Taheri T. (2017). Motor neuron transdifferentiation of neural stem cell from adipose-derived stem cell characterized by differential gene expression. *Cellular and Molecular Neurobiology*.

[12] Paíno C., Muñoz M., Barrio L., González Nieto D., Velosillo L. (2015). Myelinating oligodendrocytes generated by direct cell reprogramming from adult rat adipose tissue. *XII European Meeting on Glial Cells in Health and Disease*.

[13] Fu X., Tong Z., Li Q. (2016). Induction of adipose-derived stem cells into Schwann-like cells and observation of Schwann-like cell proliferation. *Molecular Medicine Reports*.

[14] Simonacci F., Bertozzi N., Raposio E. (2017). Off-label use of adipose-derived stem cells. *Annals of Medicine and Surgery*.

[15] Ferraro G. A., Mizuno H., Pallua N. (2016). Adipose stem cells: from bench to bedside. *Stem Cells International*.

[16] Tobita M., Tajima S., Mizuno H. (2015). Adipose tissue-derived mesenchymal stem cells and platelet-rich plasma: stem cell transplantation methods that enhance stemness. *Stem Cell Research & Therapy*.

[17] Lv F. J., Tuan R. S., Cheung K. M. C., Leung V. Y. L. (2014). Concise review: the surface markers and identity of human mesenchymal stem cells. *Stem Cells*.

[18] Pachón-Peña G., Donnelly C., Ruiz-Cañada C. (2017). A glycovariant of human CD44 is characteristically expressed on human mesenchymal stem cells. *Stem Cells*.

[19] Leibacher J., Henschler R. (2016). Biodistribution, migration and homing of systemically applied mesenchymal stem/stromal cells. *Stem Cell Research & Therapy*.

[20] Siciliano C., Bordin A., Ibrahim M. (2016). The adipose tissue of origin influences the biological potential of human adipose stromal cells isolated from mediastinal and subcutaneous fat depots. *Stem Cell Research*.

[21] Gimble J. M., Ray S. P., Zanata F. (2016). Adipose derived cells and tissues for regenerative medicine. *ACS Biomaterials Science & Engineering*.

[22] Brettschneider J., Tredici K. D., Lee V. M. Y., Trojanowski J. Q. (2015). Spreading of pathology in neurodegenerative diseases: a focus on human studies. *Nature Reviews Neuroscience*.

[23] Vincent P. H., Benedikz E., Uhlen P., Hovatta O., Sundstrom E. (2017). Expression of pluripotency markers in nonpluripotent human neural stem and progenitor cells. *Stem Cells and Development*.

[24] Tingling J. D., Bake S., Holgate R. (2013). CD24 expression identifies teratogen-sensitive fetal neural stem cell subpopulations: evidence from developmental ethanol exposure and orthotopic cell transfer models. *PloS one*.

[25] Tomellini E., Lagadec C., Polakowska R., Le Bourhis X. (2014). Role of p 75 neurotrophin receptor in stem cell biology: more than just a marker. *Cellular and Molecular Life Sciences*.

[26] Wee B., Pietras A., Ozawa T. (2016). ABCG2 regulates self-renewal and stem cell marker expression but not tumorigenicity or radiation resistance of glioma cells. *Scientific Reports*.

[27] Purushothaman A., Sugahara K., Faissner A. (2012). Chondroitin sulfate “wobble motifs” modulate maintenance and differentiation of neural stem cells and their progeny. *Journal of Biological Chemistry*.

[28] Sabelström H., Stenudd M., Frisén J. (2014). Neural stem cells in the adult spinal cord. *Experimental Neurology*.

[29] Ruggieri M., Riboldi G., Brajkovic S. (2014). Induced neural stem cells: methods of reprogramming and potential therapeutic applications. *Progress in Neurobiology*.

[30] Cairns D. M., Chwalek K., Moore Y. E. (2016). Expandable and rapidly differentiating human induced neural stem cell lines for multiple tissue engineering applications. *Stem Cell Reports*.

[31] Ring K. L., Tong L. M., Balestra M. E. (2012). Direct reprogramming of mouse and human fibroblasts into multipotent neural stem cells with a single factor. *Cell Stem Cell*.

[32] Wapinski O. L., Vierbuchen T., Qu K. (2013). Hierarchical mechanisms for direct reprogramming of fibroblasts to neurons. *Cell*.

[33] Hermann A., Gastl R., Liebau S. (2004). Efficient generation of neural stem cell-like cells from adult human bone marrow stromal cells. *Journal of Cell Science*.

[34] Kang S. K., Putnam L. A., Ylostalo J. (2004). Neurogenesis of Rhesus adipose stromal cells. *Journal of Cell Science*.

[35] Feng N., Han Q., Li J. (2014). Generation of highly purified neural stem cells from human adipose-derived mesenchymal stem cells by Sox 1 activation. *Stem Cells and Development*.

[36] Yang E., Liu N., Tang Y. (2015). Generation of neurospheres from human adipose-derived stem cells. *BioMed Research International*.

[37] Petersen E. D., Zenchak J. R., Lossia O. V., Hochgeschwender U. (2018). Neural stem cells derived directly from adipose tissue. *Stem Cells and Development*.

[38] Qin Y., Zhou C., Wang N., Yang H., Gao W. Q. (2015). Conversion of adipose tissue-derived mesenchymal stem cells to neural stem cell-like cells by a single transcription factor, Sox 2. *Cellular Reprogramming*.

[39] Zhang Y., Liu N., Tang Y. (2014). Efficient generation of neural stem cell-like cells from rat adipose derived stem cells after lentiviral transduction with green fluorescent protein. *Molecular Neurobiology*.

[40] Qu X., Liu T., Song K., Li X., Ge D. (2013). Differentiation of reprogrammed human adipose mesenchymal stem cells toward neural cells with defined transcription factors. *Biochemical and Biophysical Research Communications*.

[41] Yang Y., Ma T., Ge J. (2016). Facilitated neural differentiation of adipose tissue-derived stem cells by electrical stimulation and Nurr-1 gene transduction. *Cell Transplantation*.

[42] Razavi S., Ahmadi N., Kazemi M., Mardani M., Esfandiari E. (2012). Efficient transdifferentiation of human adipose-derived stem cells into Schwann-like cells: a promise for treatment of demyelinating diseases. *Advanced Biomedical Research*.

[43] Ying C., Hu W., Cheng B., Zheng X., Li S. (2012). Neural differentiation of rat adipose-derived stem cells in vitro. *Cellular and Molecular Neurobiology*.

[44] Tang Y., He H., Cheng N. (2014). PDGF, NT-3 and IGF-2 in combination induced transdifferentiation of muscle-derived stem cells into Schwann cell-like cells. *PloS One*.

[45] Chen J., Tang Y. X., Liu Y. M. (2012). Transplantation of adipose-derived stem cells is associated with neural differentiation and functional improvement in a rat model of intracerebral hemorrhage. *CNS Neuroscience & Therapeutics*.

[46] Razavi S., Mardani M., Kazemi M. (2013). Effect of leukemia inhibitory factor on the myelinogenic ability of Schwann-like cells induced from human adipose-derived stem cells. *Cellular and Molecular Neurobiology*.

[47] Anghileri E., Marconi S., Pignatelli A. (2008). Neuronal differentiation potential of human adipose-derived mesenchymal stem cells. *Stem Cells and Development*.

[48] Ji W., Zhang X., Ji L., Wang K., Qiu Y. (2015). Effects of brain-derived neurotrophic factor and neurotrophin-3 on the neuronal differentiation of rat adipose-derived stem cells. *Molecular Medicine Reports*.

[49] Boulland J. L., Mastrangelopoulou M., Boquest A. C. (2013). Epigenetic regulation of nestin expression during neurogenic differentiation of adipose tissue stem cells. *Stem Cells and Development*.

[50] Madhu V., Dighe A. S., Cui Q., Deal D. N. (2016). Dual inhibition of activin/nodal/TGF-*β* and BMP signaling pathways by SB431542 and dorsomorphin induces neuronal differentiation of human adipose derived stem cells. *Stem Cells International*.

[51] Cardozo A., Ielpi M., Gómez D., Argibay A. P. (2010). Differential expression of Shh and BMP signaling in the potential conversion of human adipose tissue stem cells into neuron-like cells in vitro. *Gene Expression*.

[52] Hu F., Wang X., Liang G. (2013). Effects of epidermal growth factor and basic fibroblast growth factor on the proliferation and osteogenic and neural differentiation of adipose-derived stem cells. *Cellular Reprogramming*.

[53] Bahmani L., Taha M. F., Javeri A. (2014). Coculture with embryonic stem cells improves neural differentiation of adipose tissue-derived stem cells. *Neuroscience*.

[54] Jang S., Cho H. H., Cho Y. B., Park J. S., Jeong H. S. (2010). Functional neural differentiation of human adipose tissue-derived stem cells using bFGF and forskolin. *BMC Cell Biology*.

[55] Liu Y., Zhang Z., Qin Y. (2013). A new method for Schwann-like cell differentiation of adipose derived stem cells. *Neuroscience Letters*.

[56] Ashjian P. H., Elbarbary A. S., Edmonds B. (2003). In vitro differentiation of human processed lipoaspirate cells into early neural progenitors. *Plastic and Reconstructive Surgery*.

[57] Lo Furno D., Pellitteri R., Graziano A. C. E. (2013). Differentiation of human adipose stem cells into neural phenotype by neuroblastoma- or olfactory ensheathing cells-conditioned medium. *Journal of Cellular Physiology*.

[58] Khosravizadeh Z., Sh R. (2015). Neuronal markers expression of induced human adipose-derived stem cells in alginate hydrogel. *Iranian Journal of Reproductive Medicine*.

[59] Jaatinen L., Salemi S., Miettinen S., Hyttinen J., Eberli D. (2015). The combination of electric current and copper promotes neuronal differentiation of adipose-derived stem cells. *Annual Review of Biomedical Engineering*.

[60] Cardozo A. J., Gomez D. E., Argibay P. F. (2012). Neurogenic differentiation of human adipose-derived stem cells: relevance of different signaling molecules, transcription factors, and key marker genes. *Gene*.

[61] Woodbury D., Schwarz E. J., Prockop D. J., Black I. B. (2000). Adult rat and human bone marrow stromal cells differentiate into neurons. *Journal of Neuroscience Research*.

[62] Hsueh Y. Y., Chang Y. J., Huang C. W. (2015). Synergy of endothelial and neural progenitor cells from adipose-derived stem cells to preserve neurovascular structures in rat hypoxic-ischemic brain injury. *Scientific Reports*.

[63] Romanov Y. A., Darevskaya A. N., Merzlikina N. V., Buravkova L. B. (2005). Mesenchymal stem cells from human bone marrow and adipose tissue: isolation, characterization, and differentiation potentialities. *Bulletin of Experimental Biology and Medicine*.

[64] Croft A. P., Przyborski S. A. (2006). Formation of neurons by non-neural adult stem cells: potential mechanism implicates an artifact of growth in culture. *Stem Cells*.

[65] Encinas J. M., Fitzsimons C. P. (2017). Gene regulation in adult neural stem cells. Current challenges and possible applications. *Advanced Drug Delivery Reviews*.

[66] Huang B., Li G., Jiang X. H. (2015). Fate determination in mesenchymal stem cells: a perspective from histone-modifying enzymes. *Stem Cell Research & Therapy*.

[67] Alabert C., Jasencakova Z., Groth A. (2017). Chromatin replication and histone dynamics. *DNA Replication*.

[68] Baumann K. (2015). Post-translational modifications: crotonylation versus acetylation. *Nature Reviews Molecular Cell Biology*.

[69] Luo L., Chen W. J., Yin J. Q., Xu R. X. (2017). EID3 directly associates with DNMT3A during transdifferentiation of human umbilical cord mesenchymal stem cells to NPC-like cells. *Scientific Reports*.

[70] Tiwari N., Berninger B. (2017). Transcriptional and epigenetic control of astrogliogenesis. *Essentials of Noncoding RNA in Neuroscience*.

[71] Jang S., Jeong H.-S. (2018). Histone deacetylase inhibition-mediated neuronal differentiation via the Wnt signaling pathway in human adipose tissue-derived mesenchymal stem cells. *Neuroscience Letters*.

[72] Talwadekar M., Fernandes S., Kale V., Limaye L. (2017). Valproic acid enhances the neural differentiation of human placenta derived-mesenchymal stem cells in vitro. *Journal of Tissue Engineering and Regenerative Medicine*.

[73] Furumatsu T., Tsuda M., Yoshida K. (2005). Sox 9 and p 300 cooperatively regulate chromatin-mediated transcription. *The Journal of Biological Chemistry*.

[74] Feller C., Forne I., Imhof A., Becker P. B. (2015). Global and specific responses of the histone acetylome to systematic perturbation. *Molecular Cell*.

[75] Carlberg C., Molnár F. (2018). The histone code. *Human Epigenomics*.

[76] Barski A., Cuddapah S., Cui K. (2007). High-resolution profiling of histone methylations in the human genome. *Cell*.

[77] Ozkul Y., Galderisi U. (2016). The impact of epigenetics on mesenchymal stem cell biology. *Journal of Cellular Physiology*.

[78] Hsieh J., Zhao X. (2016). Genetics and epigenetics in adult neurogenesis. *Cold Spring Harbor Perspectives in Biology*.

[79] Berdasco M., Melguizo C., Prados J. (2012). DNA methylation plasticity of human adipose-derived stem cells in lineage commitment. *The American Journal of Pathology*.

[80] Alexanian A. R. (2015). Epigenetic modulators promote mesenchymal stem cell phenotype switches. *The International Journal of Biochemistry & Cell Biology*.

[81] Saidi N., Ghalavand M., Hashemzadeh M. S., Dorostkar R., Mohammadi H., Mahdian-shakib A. (2017). Dynamic changes of epigenetic signatures during chondrogenic and adipogenic differentiation of mesenchymal stem cells. *Biomedicine & Pharmacotherapy*.

[82] Noer A., Sorensen A. L., Boquest A. C., Collas P. (2006). Stable CpG hypomethylation of adipogenic promoters in freshly isolated, cultured, and differentiated mesenchymal stem cells from adipose tissue. *Molecular Biology of the Cell*.

[83] Wang Y., Wang D., Guo D. (2016). MiR-124 promote neurogenic transdifferentiation of adipose derived mesenchymal stromal cells partly through RhoA/ROCK1, but not ROCK2 signaling pathway. *PloS One*.

[84] He X., Ao Q., Wei Y., Song J. (2016). Transplantation of miRNA-34a overexpressing adipose-derived stem cell enhances rat nerve regeneration. *Wound Repair and Regeneration*.

[85] Huang S., Wang S., Bian C. (2012). Upregulation of miR-22 promotes osteogenic differentiation and inhibits adipogenic differentiation of human adipose tissue-derived mesenchymal stem cells by repressing *HDAC6* protein expression. *Stem Cells and Development*.

[86] Lee E. K., Lee M. J., Abdelmohsen K. (2011). miR-130 suppresses adipogenesis by inhibiting peroxisome proliferator-activated receptor γ expression. *Molecular Biology of the Cell*.

[87] Yang Z., Bian C., Zhou H. (2011). MicroRNA hsa-miR-138 inhibits adipogenic differentiation of human adipose tissue-derived mesenchymal stem cells through adenovirus EID-1. *Stem Cells and Development*.

[88] Aji K., Zhang Y., Aimaiti A. (2017). MicroRNA-145 regulates the differentiation of human adipose-derived stem cells to smooth muscle cells via targeting Krüppel-like factor 4. *Molecular Medicine Reports*.

[89] Liu S., Yang Y., Wu J. (2011). TNFα-induced up-regulation of miR-155 inhibits adipogenesis by down-regulating early adipogenic transcription factors. *Biochemical and Biophysical Research Communications*.

[90] Peng Y., Li H., Li X. (2016). MicroRNA-215 impairs adipocyte differentiation and co-represses FNDC3B and CTNNBIP1. *The International Journal of Biochemistry & Cell Biology*.

[91] Peng Y., Xiang H., Chen C. (2013). MiR-224 impairs adipocyte early differentiation and regulates fatty acid metabolism. *The International Journal of Biochemistry & Cell Biology*.

[92] Bork S., Horn P., Castoldi M., Hellwig I., Ho A. D., Wagner W. (2011). Adipogenic differentiation of human mesenchymal stromal cells is down-regulated by microRNA-369-5p and up-regulated by microRNA-371. *Journal of Cellular Physiology*.

[93] Kraus M., Greither T., Wenzel C., Bräuer-Hartmann D., Wabitsch M., Behre H. M. (2015). Inhibition of adipogenic differentiation of human SGBS preadipocytes by androgen-regulated microRNA miR-375. *Molecular and Cellular Endocrinology*.

[94] Yoo A. S., Sun A. X., Li L. (2011). MicroRNA-mediated conversion of human fibroblasts to neurons. *Nature*.

[95] Delaloy C., Liu L., Lee J. A. (2010). MicroRNA-9 coordinates proliferation and migration of human embryonic stem cell-derived neural progenitors. *Cell Stem Cell*.

[96] Sun G., Ye P., Murai K. (2011). miR-137 forms a regulatory loop with nuclear receptor TLX and LSD1 in neural stem cells. *Nature Communications*.

[97] Hill M. J., Donocik J. G., Nuamah R. A., Mein C. A., Sainz-Fuertes R., Bray N. J. (2014). Transcriptional consequences of schizophrenia candidate miR-137 manipulation in human neural progenitor cells. *Schizophrenia Research*.

[98] Zhao C., Sun G., Li S. (2010). MicroRNA *let-7b* regulates neural stem cell proliferation and differentiation by targeting nuclear receptor TLX signaling. *Proceedings of the National Academy of Sciences of the United States of America*.

[99] Zhao C., Sun G. Q., Ye P., Li S., Shi Y. (2013). MicroRNA let-7d regulates the TLX/microRNA-9 cascade to control neural cell fate and neurogenesis. *Scientific Reports*.

[100] Yu K.-R., Shin J. H., Kim J. J. (2015). Rapid and efficient direct conversion of human adult somatic cells into neural stem cells by HMGA2/let-7b. *Cell Reports*.

[101] Liu C., Teng Z. Q., Santistevan N. J. (2010). Epigenetic regulation of miR-184 by MBD1 governs neural stem cell proliferation and differentiation. *Cell Stem Cell*.

[102] Lim L. P., Lau N. C., Garrett-Engele P. (2005). Microarray analysis shows that some microRNAs downregulate large numbers of target mRNAs. *Nature*.

[103] Shi Y., Zhao X., Hsieh J. (2010). MicroRNA regulation of neural stem cells and neurogenesis. *Journal of Neuroscience*.

[104] Tat P. A., Sumer H., Jones K. L., Upton K., Verma P. J. (2010). The efficient generation of induced pluripotent stem (iPS) cells from adult mouse adipose tissue-derived and neural stem cells. *Cell Transplantation*.

[105] Sun N., Panetta N. J., Gupta D. M. (2009). Feeder-free derivation of induced pluripotent stem cells from adult human adipose stem cells. *Proceedings of the National Academy of Sciences*.

[106] Bielefeld P., Schouten M., Lucassen P. J., Fitzsimons C. P. (2017). Transcription factor oscillations in neural stem cells: implications for accurate control of gene expression. *Neurogenesis*.

[107] Visweswaran M., Pohl S., Arfuso F. (2015). Multi-lineage differentiation of mesenchymal stem cells - to Wnt, or not Wnt. *The International Journal of Biochemistry & Cell Biology*.

[108] Van Camp J. K., Beckers S., Zegers D., Van Hul W. (2014). Wnt signaling and the control of human stem cell fate. *Stem Cell Reviews*.

[109] Jang S., Park J. S., Jeong H. S. (2015). Neural differentiation of human adipose tissue-derived stem cells involves activation of the Wnt5a/JNK signalling. *Stem Cells International*.

[110] Bizen N., Inoue T., Shimizu T., Tabu K., Kagawa T., Taga T. (2014). A growth-promoting signaling component cyclin D1 in neural stem cells has antiastrogliogenic function to execute self-renewal. *Stem Cells*.

[111] Kléber M., Lee H.-Y., Wurdak H. (2005). Neural crest stem cell maintenance by combinatorial Wnt and BMP signaling. *The Journal of Cell Biology*.

[112] Bowman A. N., van Amerongen R., Palmer T. D., Nusse R. (2013). Lineage tracing with Axin2 reveals distinct developmental and adult populations of Wnt/β-catenin–responsive neural stem cells. *Proceedings of the National Academy of Sciences of the United States of America*.

[113] Nusse R., Clevers H. (2017). Wnt/β-catenin signaling, disease, and emerging therapeutic modalities. *Cell*.

[114] Wisniewska M. B. (2013). Physiological role of β-catenin/TCF signaling in neurons of the adult brain. *Neurochemical Research*.

[115] Inestrosa N. C., Varela-Nallar L. (2015). Wnt signalling in neuronal differentiation and development. *Cell and Tissue Research*.

[116] Bray S. J. (2016). Notch signalling in context. *Nature Reviews Molecular Cell Biology*.

[117] Osathanon T., Subbalekha K., Sastravaha P., Pavasant P. (2012). Notch signalling inhibits the adipogenic differentiation of single-cell-derived mesenchymal stem cell clones isolated from human adipose tissue. *Cell Biology International*.

[118] Kingham P. J., Mantovani C., Terenghi G. (2009). Notch independent signalling mediates Schwann cell-like differentiation of adipose derived stem cells. *Neuroscience Letters*.

[119] Cardozo A. J., Gómez D. E., Argibay P. F. (2011). Transcriptional characterization of Wnt and Notch signaling pathways in neuronal differentiation of human adipose tissue-derived stem cells. *Journal of Molecular Neuroscience*.

[120] Jing W., Xiong Z., Cai X. (2010). Effects of γ-secretase inhibition on the proliferation and vitamin D 3 induced osteogenesis in adipose derived stem cells. *Biochemical and Biophysical Research Communications*.

[121] Venkatesh K., Reddy L. V. K., Abbas S. (2017). NOTCH signaling is essential for maturation, self-renewal, and tri-differentiation of *in vitro* derived human neural stem cells. *Cellular Reprogramming*.

[122] Ludwig P. E., Thankam F. G., Patil A. A., Chamczuk A. J., Agrawal D. K. (2018). Brain injury and neural stem cells. *Neural Regeneration Research*.

[123] Meyers E. A., Kessler J. A. (2017). TGF-β family signaling in neural and neuronal differentiation, development, and function. *Cold Spring Harbor Perspectives in Biology*.

[124] Kakudo N., Kushida S., Suzuki K. (2012). Effects of transforming growth factor-beta 1 on cell motility, collagen gel contraction, myofibroblastic differentiation, and extracellular matrix expression of human adipose-derived stem cell. *Human Cell*.

[125] Chambers S. M., Mica Y., Lee G., Studer L., Tomishima M. J. (2013). Dual-SMAD inhibition/WNT activation-based methods to induce neural crest and derivatives from human pluripotent stem cells. *Human Embryonic Stem Cell Protocols*.

[126] Hu J. G., Zhang Y. X., Qi Q. (2012). Expression of BMP-2 and BMP-4 proteins by type-1 and type-2 astrocytes induced from neural stem cells under different differentiation conditions. *Acta Neurobiologiae Experimentalis*.

[127] Jovanovic V. M., Salti A., Tilleman H. (2018). BMP/SMAD pathway promotes neurogenesis of midbrain dopaminergic neurons *in vivo* and in human induced pluripotent and neural stem cells. *The Journal of Neuroscience*.

[128] De Almeida I., Oliveira N. M. M., Randall R. A., Hill C. S., McCoy J. M., Stern C. D. (2017). Calreticulin is a secreted BMP antagonist, expressed in Hensen’s node during neural induction. *Developmental Biology*.

[129] Xu F. T., Li H. M., Yin Q. S. (2014). Effect of ginsenoside Rg1 on proliferation and neural phenotype differentiation of human adipose-derived stem cells in vitro. *Canadian Journal of Physiology and Pharmacology*.

[130] Liqing Y., Jia G., Jiqing C. (2011). Directed differentiation of motor neuron cell-like cells from human adipose-derived stem cells in vitro. *Neuroreport*.

[131] Llorens-Bobadilla E., Martin-Villalba A. (2017). Adult NSC diversity and plasticity: the role of the niche. *Current Opinion in Neurobiology*.

[132] Blecker D., Elashry M. I., Heimann M., Wenisch S., Arnhold S. (2017). New insights into the neural differentiation potential of canine adipose tissue-derived mesenchymal stem cells. *Anatomia, Histologia, Embryologia*.

[133] Arribas M. I., Ropero A. B., Reig J. A. (2014). Negative neuronal differentiation of human adipose-derived stem cell clones. *Regenerative Medicine*.

[134] Heng B. C., Saxena P., Fussenegger M. (2014). Heterogeneity of baseline neural marker expression by undifferentiated mesenchymal stem cells may be correlated to donor age. *Journal of Biotechnology*.

